# Exploring the Metabolism of Flubrotizolam, a Potent Thieno-Triazolo Diazepine, Using Human Hepatocytes and High-Resolution Mass Spectrometry

**DOI:** 10.3390/metabo14090506

**Published:** 2024-09-19

**Authors:** Prince Sellase Gameli, Johannes Kutzler, Diletta Berardinelli, Jeremy Carlier, Volker Auwärter, Francesco Paolo Busardò

**Affiliations:** 1 Section of Legal Medicine, Department of Biomedical Sciences and Public Health, Marche Polytechnic University, 60126 Ancona, Italy; p.s.gameli@pm.univpm.it (P.S.G.); d.berardinelli@pm.univpm.it (D.B.); f.p.busardo@staff.univpm.it (F.P.B.); 2 Institute of Forensic Medicine, Medical Center-University of Freiburg, Faculty of Medicine, University of Freiburg, 79104 Freiburg, Germany; johannes.kutzler@uniklinik-freiburg.de (J.K.); volker.auwaerter@uniklinik-freiburg.de (V.A.)

**Keywords:** designer benzodiazepine, new psychoactive substances, metabolism, high-resolution mass spectrometry, biomarker

## Abstract

Background: The abuse of psychoactive substances presents challenges in clinical and forensic toxicology. The emergence of novel and potent drugs that pose significant health risks, in particular towards frequent abusers and users unaware of the ingredients, further complicates the situation. Designer benzodiazepines have become a fast-growing subgroup of these new psychoactive substances (NPSs), and their overdose may potentially turn fatal, especially when combined with other central nervous system depressants. In 2021, flubrotizolam, a potent thieno-triazolo designer benzodiazepine, emerged on the illicit market, available online as a “research chemical”. The identification of markers of consumption for this designer benzodiazepine is essential in analytical toxicology, especially in clinical and forensic cases. Methods: We therefore aimed to identify biomarkers of flubrotizolam uptake in ten-donor-pooled human hepatocytes, applying liquid chromatography high-resolution mass spectrometry and software-aided data mining supported by in silico prediction tools. Results: Prediction studies resulted in 10 and 13 first- and second-generation metabolites, respectively, mainly transformed through hydroxylation and sulfation, methylation, and glucuronidation reactions. We identified six metabolites after 3 h human hepatocyte incubation: two hydroxylated metabolites (α- and 6-hydroxy-flubrotizolam), two 6-hydroxy-glucuronides, a reduced-hydroxy-*N*-glucuronide, and an *N*-glucuronide. Conclusions: We suggest detecting flubrotizolam and its hydroxylated metabolites as markers of consumption after the glucuronide hydrolysis of biological samples. The results are consistent with the in vivo metabolism of brotizolam, a medically used benzodiazepine and a chloro-phenyl analog of flubrotizolam.

## 1. Introduction

Benzodiazepine prescription and use have become essential in contemporary medicine as relatively safe anxiolytics. Nonetheless, their potential to produce addiction has raised serious health concerns. This is further exacerbated by the growing number of synthetic or designer benzodiazepines appearing on the market [1]. Designer benzodiazepines are a subgroup of new psychoactive substances (NPSs), which are compounds not controlled by the 1961 Single Convention on Narcotic Drugs or the 1971 Convention on Psychotropic Substances. These NPSs first emerged more than a decade ago in Europe and include structural variations of known benzodiazepines (e.g., pyrazolam), as well as pharmacologically active metabolites like fonazepam. They are largely sold on various online platforms and marked as “research chemicals” or “legal highs”, or they are intentionally misbranded as approved medications (e.g., fake Xanax^®^) [2,3]. Presently, more than 50 designer benzodiazepines are monitored by various national and international agencies, and legislative changes are constantly evolving to address legal loopholes that enable the proliferation of not only designer benzodiazepines but of NPSs more generally [4,5].

Flubrotizolam (2-bromo-4-(2′-fluorophenyl)-9-methyl-6*H*-thieno [3,2-f][1,2,4]triazolo [4,3-a][1,4]diazepine), also known as “fanax” or “JYI-73”, is a thieno-triazolo designer benzodiazepine structurally similar to brotizolam (Lendormin^®^) that carries a chlorine instead of a fluorine at position 2 of the phenyl ring. The drug can be prescribed as a sedative in Japan and some parts of Europe [6,7]. Catalani et al., 2023, reported on the extraordinarily high biological activity for flubrotizolam in an in silico quantitative structure–activity relationship (QSAR) study, though in vivo studies will be necessary in order to substantiate this finding [8,9,10].

Flubrotizolam was first reported in Denmark in 2021 and has been reported in cases of people driving under the influence of drugs in Europe, Canada, New Zealand, and the USA according to the United Nations Office on Drugs and Crime (Early-Warning System) and the European Union Drugs Agency, formerly known as the European Monitoring Centre for Drugs and Drug Addiction [11,12]. Considering flubrotizolam’s presumed high potency, it is likely to be concomitantly abused with other drugs, especially opioids to enhance the combined synergistic and inebriating effects, as seen in the recent “benzo-dope” phenomenon [13], or as “standby medication” for hallucinogen users [14]. There are limited data on the toxidromes associated with flubrotizolam intoxication, and very little is known on the metabolism of this designer benzodiazepine. Less than 1% of dosed brotizolam is excreted unchanged in urine, with the remainder being primarily excreted as hydroxylated and conjugated metabolites, and considering their structural resemblance, flubrotizolam might follow the same pattern [15]. Taking into account flubrotizolam’s likely extensive hepatic metabolism, and therefore their challenging detection in biological matrices, it is pertinent to identify consumption markers by targeting metabolites which may have extended windows of detection. Metabolite profiling via in vitro models such human hepatocytes, with adequate metabolic enzymes, cofactors, and transporters, has proven to accurately simulate phases I and II metabolism of drugs and xenobiotics in humans [16,17,18,19]. This combined with in silico prediction tools and high-resolution mass-spectrometry-based metabolomics present an invaluable arsenal for clinical and analytical toxicologists in improving the detection of authentic positive cases [20].

The present study therefore aims to investigate flubrotizolam metabolism by employing web-based in silico prediction tools in conjunction with in vitro incubation with human hepatocytes pooled from ten donors. Analysis was performed with ultra-high-performance liquid chromatography–high-resolution tandem mass spectrometry (UHPLC-HRMS/MS) with software-assisted data mining tools to improve the detection of flubrotizolam in clinical and forensic cases.

## 2. Materials and Methods

### 2.1. Chemicals and Materials

The pure standard of flubrotizolam was provided within the framework of the EU-project ADEBAR plus [21], and diclofenac (experimental control) was obtained from Sigma Aldrich (Milan, Italy). Stock solutions at 1 mg/mL were prepared in methanol and stored at −20 °C. LC-MS-grade acetonitrile, water, methanol, and formic acid were purchased from Carlo Erba (Cornaredo, Italy) and reagent-grade ammonium acetate was obtained from Levanchimica (Bari, Italy). Cryopreserved human hepatocytes (pooled from ten donors), thawing medium, and trypan blue (0.4%) were obtained from Lonza (Basel, Switzerland). Williams’ medium E (WME), *l*-glutamine, and HEPES buffer (2-[4-(2-hydroxyethyl)-1-piperazinyl] ethanesulfonic acid) were also procured from Sigma Aldrich. HEPES and *l*-glutamine, 2 and 20 mmol/L, respectively, were used in preparing Supplemented Williams’ Medium E (SWM) and stored at 4 °C until incubation. Finally, β-glucuronidase from *Patella vulgata* L. was obtained from Sigma Aldrich and used for hydrolysis.

### 2.2. In Silico Metabolite Prediction

Metabolites of flubrotizolam were predicted using GLORYx, a web-based machine learning and prediction model, to simulate phase I and phase II metabolic reactions in humans. A simplified molecular input line entry system (SMILES), generated using ACD/ChemSketch (Freeware; v. 2020.1.2), was used in the “phase I and phase II metabolism” option in GLORYx [22,23]. First- and second-generation metabolites, metabolites following one and two biotransformational reactions, respectively, with a probability score equal to or greater than 20%, were then incorporated in an inclusion list for LC-HRMS/MS analysis (Section 2.5.2).

### 2.3. Incubation with Pooled Human Hepatocytes

Flubrotizolam was incubated with cryopreserved human hepatocytes using a previously described protocol with marginal changes where necessary [24,25]. To summarize, hepatocytes were thawed in 50 mL of thawing medium, centrifuged for 5 min at 50–100× *g*, and the pellets resuspended in 50 mL thawing medium. The pellets were resuspended in 2 mL of SWM after the supernatant was discarded following a second centrifugation under the same conditions. The cell viability was then assessed using the Trypan blue exclusion method and the volume of SWM was adjusted to 2 × 10^6^ viable cells/mL. Then, 250 μL of hepatocyte suspension was gently mixed with 250 μL of 20 μmol/L flubrotizolam in SWM in a sterile 24-well culture plate using an ArgoLab incubator (Arezzo, Italy). Diclofenac (used as a positive control) and negative controls were incubated alongside. The reactions were quenched with 500 μL of ice-cold acetonitrile, centrifuged for 10 min at 15,000× *g* and stored at −80 °C for further analysis the following day. All reactions were conducted under physiological conditions.

### 2.4. Sample Preparation

#### 2.4.1. Human Hepatocyte Incubates

The incubates that thawed at room temperature were centrifuged at 15,000× *g* for 10 min, and 100 μL of the supernatant was mixed with 100 μL of acetonitrile and centrifuged again at 15,000× *g* for 10 min. The supernatant was evaporated to dryness under nitrogen at 37 °C and the remaining residue was reconstituted in 100 μL of mobile phases A:B (MPA:B, 90:10, *v*/*v*; see Section 2.5.1). After vortexing, the resulting mixture was transferred into a glass vial and 10 μL was injected once in positive and negative ionization mode for LC-HRMS/MS data acquisition.

#### 2.4.2. β-Glucuronidase Hydrolysis

To clarify the elucidated structure of specific glucuronide conjugates, we re-analyzed the 3 h hepatocyte incubation with flubrotizolam after hydrolysis.

The incubate was thawed at room temperature and centrifuged for 10 min at 15,000× *g*, and 50 µL of the supernatant was incubated with 50 µL of β-glucuronidase (*P. vulgata*) and 5 µL of 10 mol/L ammonium acetate under pH 5 conditions at 37 °C for 90 min. After hydrolysis, 200 µL of acetonitrile was added and the mixture was centrifuged at 15,000× *g* for 10 min and evaporated to dryness under nitrogen. The remaining residue was reconstituted with MPA:B (90:10 *v*/*v*) and centrifuged again, and 10 µL of the supernatant was injected in the LC-HRMS/MS for analysis.

An experimental control with 3 h hepatocyte incubation was performed with water (instead of alongside β-glucuronidase) to monitor the reaction and ensure the reaction was enzymatic.

### 2.5. Instrumental Conditions

LC-HRMS/MS (from Thermo Scientific (Waltham, MA, USA), equipped with UHPLC, a DIONEX UtiMate 3000 chromatography system, and Q-Exactive quadrupole–orbitrap hybrid high-resolution mass spectrometry with a heated electrospray ionization (HESI) source, was used to acquire the data. The mass spectrometer was operated in positive and negative ionization modes.

#### 2.5.1. Liquid Chromatography

A Kinetex^®^ Biphenyl column (150 × 2.1 mm, 2 μm) from Phenomenex (Torrance, CA, USA), maintained at 37 °C, was used for the chromatographic run. Mobile phases A and B consisted of 0.1% formic acid in water and 0.1% formic acid in acetonitrile, respectively. The separation was performed at a flow rate of 0.4 mL/min for 25 min. The gradient elution of MPA:B (98:2) was maintained for the first 2 min and B was gradually increased to 45% and 95% within 14.5 and 1 min, respectively. MPB was maintained at 95% till 20.5 min, and the conditions at the start were then restored within 0.1 min and maintained till the end of the run.

#### 2.5.2. Mass Spectrometry

The HESI source settings of normalized collision energy (NCE) were optimized with 1 μg/mL of flubrotizolam standard in MPA:B (90:10, *v*/*v*). The HESI settings were as follows: a spray voltage of 3.5 kV; capillary and auxiliary temperatures of 300 °C; sheath and auxiliary gas flow rates of 5 and 50 AU, respectively; and a S-lens frequency of 50. The orbitrap was fully calibrated before the analysis, with a lock mass list used for better accuracy. Data acquisition was performed from 1 to 18 min in full-scan MS and data-dependent MS/MS modes. The automatic gain control (AGC) target in full-scan MS mode was 1 × 10^6^ with a resolution of 70,000 at full width at half maximum at *m*/*z* 200. The maximum injection time (IT) was 256 ms and the scan range in positive and negative ionization modes was *m*/*z* 200–700. The settings for the data-dependent MS/MS included an AGC target of 2 × 10^5^ with a minimum target of 6.5 × 10^2^, a resolution of 17,500, a maximum IT of 64 ms, and an isolation window of *m*/*z* 1.2. The NCE was 30, 50, and 70 AU with a loop count of 5 and a dynamic exclusion of 2.0 s. An inclusion list was added for the data-dependent acquisition mode. Two different inclusion lists were compiled using ^79^Br and ^81^Br in order to obtain non-interfered MS^2^ spectra due to the isotopic distribution of flubrotizolam. The inclusion lists of all plausible transformations for MS/MS acquisition are included in Supplementary Table S1.

### 2.6. Identification of Metabolites

The data from LC-HRMS/MS were processed with Compound Discoverer (v. 3.1.1.12) from Thermo Scientific (Waltham, MA, USA) by remodeling a previously elaborated approach [25]. The workflow utilizing a targeted and non-targeted approach is as follows: the ions detected in HRMS/MS were compared to theoretically generated metabolites, as shown in Supplementary Table S2 (intensity threshold: 5 × 10^3^; mass tolerance: 5 ppm). The HRMS/MS spectra and elucidated elemental composition of the predicted and unpredicted metabolites were compared with mzCloud (databases of counterfeit drugs, drugs of abuse/illegal drugs, and prescription drugs) and ChemSpider (Cayman Chemical and DrugBank, with an intensity threshold of 10^5^) with mass tolerance at 5 and 10 ppm, respectively. All predicted metabolites, potential reactions, and workflow are included in Supplementary Table S2.

## 3. Results

### 3.1. In Silico Prediction

Ten first-generation (pM1–pM10, by decreasing probability score) and thirteen second-generation (pMX-1–pMX-13, by decreasing score, with pMX representing the corresponding first-generation metabolite) metabolites were produced using GLORYx freeware at a 20% threshold limit. Hydroxylation, *N*-dealkylation, and *N*-oxidation reactions occurring at the diazepine substructure were the highest predicted metabolites (40%). Phenyl hydroxylation and subsequent phase II glucuronidation, sulfation, and methylation reactions were the most prevalent metabolites predicted in silico. Other predicted transformations included hydroxylation at the pyrazole ring, carboxylation, sulfide oxidation, and glutathionylation. The predicted transformations, elemental composition, probability score, and SMILES of all predicted metabolites are detailed in Supplementary Table S3.

### 3.2. Flubrotizolam HRMS/MS Fragmentation

Ramped collision energies were employed in positive-ionization mode to produce desirable fragments and elucidate the chemical structures of flubrotizolam and its metabolites. Under the present analytical conditions, flubrotizolam did not produce a signal in the negative ionization mode when 1 μg/mL of the standard was injected in MPA:B (90:10, *v*/*v*); the same source conditions and NCE were therefore used in both ionization modes. In this article, MS and MS/MS spectra are described in positive ionization mode unless specified otherwise.

**Figure 1:**
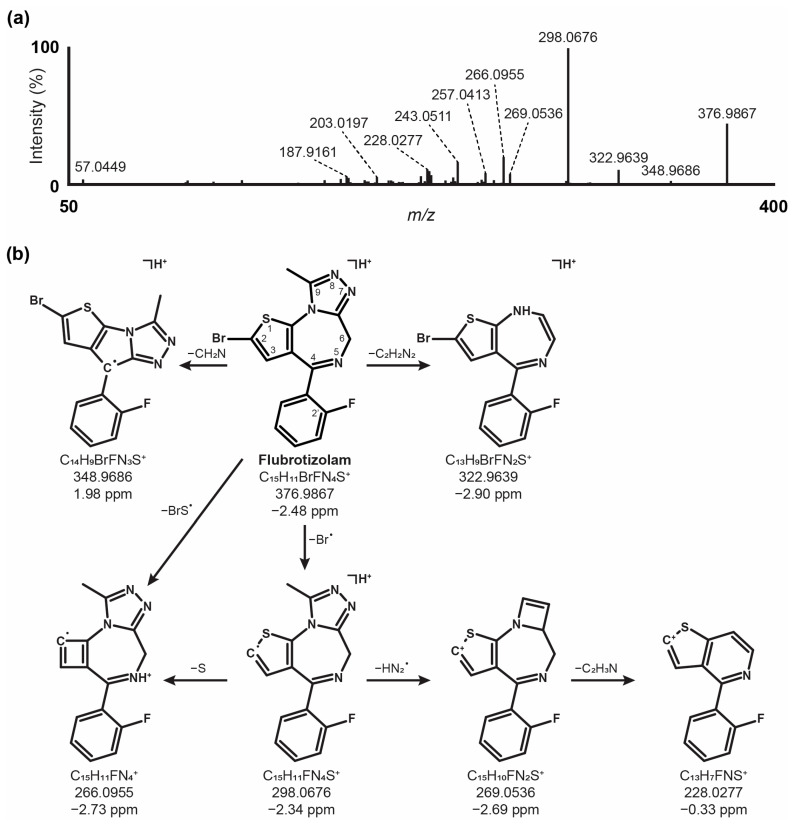
HRMS/MS fragmentation spectrum (**a**) and proposed fragmentation pathway of flubrotizolam (**b**).

Flubrotizolam was detected at 15.08 min and showed a signal at *m*/*z* 376.9862. The fragmentation spectrum and proposed fragmentation pattern after 3 h of incubation and analysis with LC-HRMS/MS in positive ionization modes are shown in Figure 1. Tentative fragment identification was based on basic MS rules, known as gas-phase reactions, as well as data from the literature [26]. The fragmentation pathway depictions (see Supplementary Figure S1 for details) are exemplary and do not necessarily reflect the real consecutive fragmentation steps. The base peak at *m*/*z* 298.0676 (C_15_H_11_FN_4_S^+●^, −2.3 ppm) can be interpreted as the result of a homolytic cleavage of the bromine atom from the parent ion. A subsequent 1,4-hydrogen shift and a loss of N_2_ (*m*/*z* 270.0621, not observed) in combination with a loss of hydrogen cyanide could lead to the fragment at *m*/*z* 243.0511 (C_14_H_10_FNS^+●^, −0.6 ppm). This contraction of flubrotizolam‘s diazepine ring has also been described for other benzodiazepines [27]. The loss of a bromosulfanyl radical from the parent molecule leads to *m*/*z* 266.0955 (C_15_H_11_FN_4_^+●^, −2.7 ppm). This transition could also be adapted for an equivalent fragment at *m*/*z* 282.0667 (C_15_H_11_ClN_4_^+●^, −0.1 ppm) of the chloro derivative, and brotizolam from spectrum data from the HighResNPS database [28]. Other fragment ions likely correspond to the loss of N_2_ (*m*/*z* 348.9804, not observed) in combination with the loss of hydrogen bromide (*m*/*z* 269.0536, C_15_H_10_FN_2_S^+^, −2.7 ppm) followed by the loss of ethenimine (*m*/*z* 228.0277, C_13_H_7_FNS^+^, −0.3 ppm). Additionally, the non-observed [M + H − N_2_]^+^ fragment could also undergo a 1,6-hydrogen shift and a subsequent loss of ethylene to result in *m*/*z* 322.9639 (C_13_H_9_BrFN_4_S^+●^, −3.0 ppm). Starting from this fragment, two major fragment pathways are likely. First, a cleavage of the fluorophenyl ring could yield *m*/*z* 226.9267 (C_7_H_4_BrN_2_S^+^, −2.7 ppm), which could undergo a further loss of hydrogen cyanide (*m*/*z* 187.9161, C_5_H_3_BrNS^+^, −1.6 ppm), followed by the potential cleavage of a bromine radical to form the fragment at *m*/*z* 108.9979 (C_5_H_3_NS^+●^, −1.6 ppm). Second, the [M + H − N_2_ − C_2_H_2_]^+^ fragment could lose a bromomethyl radical (*m*/*z* 230.0300, C_12_H_7_FN_2_S^+●^, −3.7 ppm) and subsequently hydrogen cyanide (*m*/*z* 203.0197, C_11_H_6_FNS^+●^, −1.2 ppm). The final two α-cleavage steps could yield a protonated 2-fluorobenzonitrile (*m*/*z* 122.0399, C_7_H_5_FN^+^, −1.3 ppm). The parent [M + H]^+^ could also break down to *m*/*z* 349.9757 (not observed) via hydrogen cyanide loss and could then either lose a bromomethyl radical (*m*/*z* 257.0413, C_13_H_8_FN_3_S^+●^, −1.7 ppm) or a hydrogen radical by α-cleavage (*m*/*z* 348.9686, C_14_H_9_BrFN_3_S^+●^, 1.9 ppm). Out of the 14 most intense fragment masses, it was not possible to elucidate a reasonable mechanism of formation or fragment structure for *m*/*z* 241.0220, 229.0196, and 225.0691.

### 3.3. Metabolite Identification

**Figure 2:**
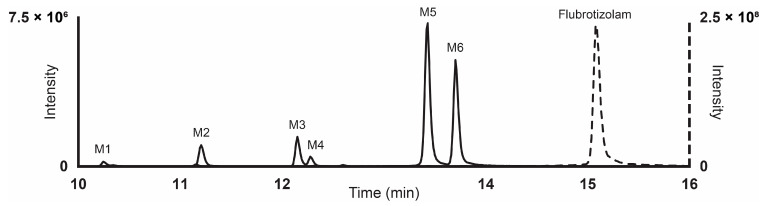
Ultra-high-performance liquid chromatography elution of flubrotizolam and its biotransformational products following 3 h of incubation with human hepatocytes. Results are shown as an extracted ion chromatogram in high-resolution mass spectrometry at the accurate mass of the compounds with a 5 ppm tolerance.

**Figure 3:**
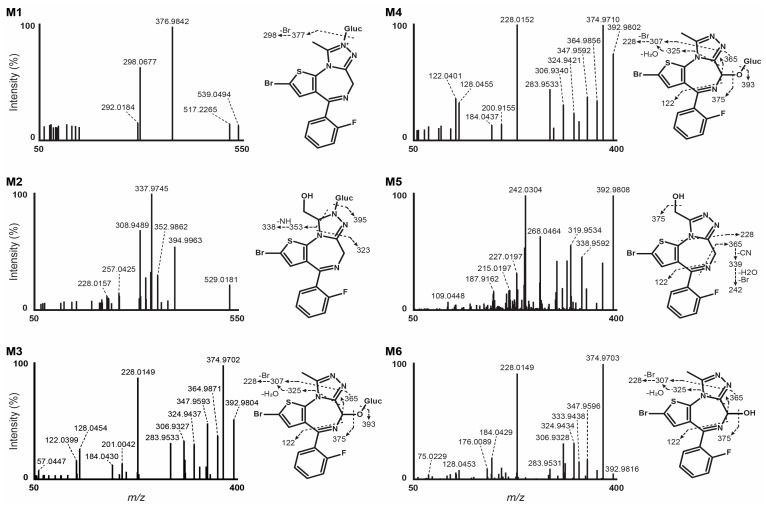
Data-dependent HRMS/MS spectra of flubrotizolam metabolites tentatively identified in hepatocyte incubations (Gluc, glucuronide). For a more detailed assignment of fragment masses to proposed structures, see Supplementary Figures S1–S4.

**Figure 4:**
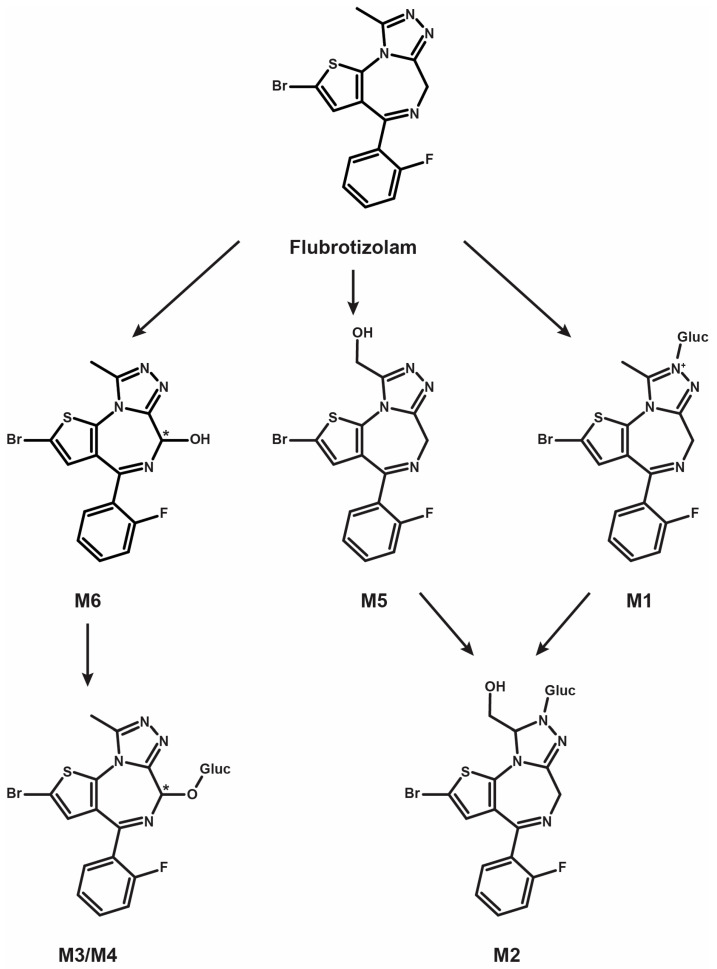
Proposed metabolic fate of the designer benzodiazepine, flubrotizolam (Gluc, glucuronide). M3 and M4 are most likely diastereomers. * denotes chiral center.

Results from LC-HRMS/MS acquisition were semi-automatically processed with the Compound Discoverer (v3.1.1.12) and yielded more than 200 plausible biotransformational products which were manually evaluated. Table 1 reports the elemental compositions, retention times, accurate masses of the molecular ions, and peak areas of flubrotizolam and metabolites in positive and negative ionization modes, where applicable, after 3 h of incubation with human hepatocytes. The peak areas of flubrotizolam after 0 and 3 h of incubation with human hepatocytes were relatively comparable, suggesting that flubrotizolam may not undergo extensive hepatic metabolism. We tentatively identified and characterized two phase I metabolites and four phase II metabolites, named M1–M6, based on the ascending order of the chromatographic elution depicted in Figure 2. The phase I metabolites, M6 and M5, are most likely hydroxylated at the diazepine and pyrazole ring, respectively, whereas the *O*-glucuronides of M6 results in M3 and M4. M6 likely exists in two stereoisomeric forms, probably not separated under the applied chromatographic conditions, whereas their *O*-glucuronides seem to be separated. A similar observation was reported in the case of 4-hydroxymidazolam [29], reduced hydroxy-*N*-glucuronide (M2), and direct *N*-glucuronide (M1), which were the phase II metabolites tentatively identified from our in vitro study. The HRMS/MS spectra of flubrotizolam metabolites and the proposed metabolic fate are presented in Figure 3 and Figure 4, respectively.

#### 3.3.1. Phase I Metabolites

We detected M5 and M6 at 13.43 and 13.70 min, respectively, with the highest peak areas among the metabolites. M5 and M6 signals were detected at *m*/*z* 392.9816 in positive ionization mode with a +15.9950 Da mass shift from that of flubrotizolam, indicating oxidation (+O). According to previous studies on alprazolam, triazolam, midazolam, and flubromazolam, which are di- or triazolo benzodiazepines similar to flubrotizolam, the 6- and α-positions are the most likely candidates for hydroxylation. Additionally, these main metabolites are also found for the chloro-derivative brotizolam [27,30,31,32].

The HRMS/MS spectrum of M6 demonstrated a base peak at *m*/*z* 374.9703, a mass shift of −18.0110 Da ([M − H_2_O]) corresponding to water loss. The fragment at *m*/*z* 228.0149 was relatively prominent (about 92% of the base peak) and could be the result of two major fragment pathways. On the one hand, M6 could lose carbon monoxide (*m*/*z* 364.9857, C_14_H_11_BrFN_4_S^+^, −2.6 ppm), enabled by a possible preceding keto-enol tautomerism [33]. This reaction is more likely to occur at a tertiary carbon atom compared to a secondary carbon atom. Therefore, this could indicate hydroxylation at the 6-position rather than the α-position, representing a secondary carbon atom. The loss of N_2_ (*m*/*z* 336.9804, not observed) and ethylene (*m*/*z* 308.9488, C_12_H_7_BrFN_2_S^+^, −1.3 ppm) combined with two consecutive α-cleavage reactions of a hydrogen radical possibly form the fragment at *m*/*z* 306.9328 (C_12_H_5_BrFN_2_S^+^, −2.4 ppm). Finally, the additional loss of a bromine radical could yield the main fragment of M6 (*m*/*z* 228.0149, C_12_H_5_FN_2_S^+●^, −1.3 ppm). On the other hand, a further potential fragmentation pathway proceeds via an elimination of N_2_ (*m*/*z* 364.9754, not observed) and prop-1-yne (*m*/*z* 324.9434, C_12_H_7_BrFN_2_OS^+^, −2.1 ppm), and could then follow the aforementioned pathway via a loss of a bromine radical. The presence of an oxygen atom in the fragment at *m*/*z* 324.9434 (possible formula C_12_H_7_BrFN_2_OS^+^) and the potential elimination of most parts of the triazole ring in the aforementioned reaction, including the α carbon, could be an indicator for the hydroxy group being located at the 6-position. An additional indicator for M6 being a 6-hydroxy metabolite is the fragment at *m*/*z* 309.9326, (C_12_H_6_BrFNOS^+^, −1.9 ppm), which could be the result of a subsequent loss of N_2_, hydrogen cyanide and ethylene. Out of the 19 most intense fragment masses, it was not possible to elucidate a reasonable formation and fragment structure for *m*/*z* 333.9438, 208.0088, and 128.0453.

The HRMS/MS spectrum of M5 substantially differs from that of M6, indicating a different position of hydroxylation. An indication of the hydroxy group being located at the α-position can be found at *m*/*z* 364.9624 (C_14_H_9_BrFN_3_OS^+●^, −1.2 ppm). Similar to several other benzodiazepines, the elimination of hydrogen cyanide is associated with a contraction of the 1,4-diazepine ring. With an additional loss of a hydrogen radical, the result of this elimination could likely be the fragment at *m*/*z* 364.9624, also eliminating the carbon at position 6. The same fragmentation pathway was observed in the parent’s HRMS/MS spectrum yielding C_14_H_9_BrFN_3_S^+^ (*m*/*z* 348.9686, 1.9 ppm), as displayed in Figure 1. Since a reasonable formula for *m*/*z* 364.9624 is only C_14_H_9_BrFN_3_OS^+^, an oxygen atom is still present, which would be in agreement with hydroxylation at the α-position. The subsequent loss of nitrilomethyl yields *m*/*z* 338.9592 (C_13_H_9_BrFN_2_OS^+^, −1.6 ppm), which could also misleadingly be interpreted as a fragment of a 6-hydroxy metabolite. An alternative generation could run via N_2_ (*m*/*z* 364.9754, not observed) and acetylene elimination. Combined with an inclusion of the hydroxypropylene chain into the ring, this loss of acetylene could likely occur at the diazepine ring, resulting in its contraction. Equally to the [M − HCN]^+•^ fragment, the elimination of acetylene yielding the [M + H − N_2_ − C_2_H_2_]^+^ fragment means a loss of the carbon at position 6 as a consequence. The presence of an oxygen atom in the most probable formula for *m*/*z* 338.9592, C_13_H_9_BrFN_2_OS^+^, therefore appeared to be more reasonably connected to the α position. Additionally, equivalent fragments of α-OH-flubromazolam, α-OH-alprazolam, and α-OH-triazolam were reported by Wohlfarth et al. in 2017 (see Supplementary Figure S5) [27]. Out of the 14 most intense fragment masses, it was not possible to elucidate a reasonable formation and fragment structure for *m*/*z* 305.9377. A graphic overview of the discussed fragmentation pathways of M6 and M5 can be found in Supplementary Figures S2 and S3, respectively. An unambiguous structure identification of the hydroxy metabolites could be achieved using nuclear magnetic resonance (NMR) spectroscopy. Further insights into the fragment pathways could be gained from multi-stage ion-trap LC-MS^n^, the use of heavy-mass labeled reference standards, electron spin resonance (ESR), or trapping experiments with suitable reagents.

#### 3.3.2. Phase II Metabolites

We detected four phase II metabolites after glucuronide conjugation, two metabolites following *O*-glucuronidation (M4 and M3), and two *N*-glucuronide conjugates (M1 and M2). M4 and M3 had retention times of 12.28 and 12.15 min, respectively, and a molecular mass of *m*/*z* 569.0138 with a +192.0277 mass shift from flubrotizolam, indicating hydroxylation (+15.9950, +O) and further glucuronide conjugation (+176.0322, +C_6_H_8_O_6_). Similarities in the MS/MS spectra of M3 and M4 are depicted in Figure 3. Based on the chromatographic resolution and similarities of the HRMS spectra of M4 and M3, we can conclude that they are likely diastereomers of M6 (see also explanation under Section 3.3).

The fragmentation patterns of M4 and M3 were similar to that of M6, pointing towards the formation of two 6-hydroxy glucuronides. Moreover, both conjugated metabolites were completely hydrolyzed with β-glucuronidase, while the M6 signal approximately doubled, further indicating glucuronidation at the 6-hydroxy group. Therefore, the two metabolites are most likely diastereomers formed by the glucuronidation of the (6R)- and the (6S)-hydroxy metabolites (see Figure 4 for the proposed metabolism pathway).

M2 was detected at *m*/*z* 571.0294 and eluted at 11.21 min. The observed mass shift was +194.0437 Da from the parent, pointing to a combination of reduction (+2H), hydroxylation (+O), and glucuronidation (+176.0322, +C_6_H_8_O_6_). The loss of ketene forming the fragment at *m*/*z* 529.0181 (C_19_H_19_BrFN_4_O_6_S^+^, 1.1 ppm) could occur from the side chain of the triazole ring, resulting in ring opening. The breakdown of the glucuronide ring could form *m*/*z* 394.9963 (C_15_H_13_BrFN_4_OS^+^, 2.3 ppm). A further nitrogen–nitrogen bond break could yield the base peak detected at *m*/*z* 337.9745 (C_13_H_10_BrFN_3_S^+^, −3.7 ppm,) solidifying that hydroxylation did not occur at the 6-position. An intense fragment at *m*/*z* 308.9498 (C_12_H_7_BrFN_2_S^+^, 1.0 ppm) could be the result of a hydrogen cyanide loss from the base peak, leading to the contraction of the 1,4-diazepine ring (*m*/*z* 310.9652, C_12_H_9_BrFN_2_S^+^, −1.3 ppm) and a subsequent loss of H_2_. We did not identify a reduced hydroxylated metabolite even after manual inspection, and hydrolysis with β-glucuronidase was inefficient in cleaving the glucuronide. We thus propose that M2 is a reduced hydroxy-*N*-glucuronide metabolite. A more detailed fragmentation pathway hypothesis for M2 can be found in the Supplementary Figure S4.

M1 was the earliest eluting metabolite detected with a retention time of 10.24 min and detected at *m*/*z* 553.0195 with a mass shift of + 176.0330 Da (+C_6_H_8_O_6_) from the parent molecule, indicating glucuronidation. The fragmentation pattern was poor due to low intensity, with a base peak detected at *m*/*z* 376.9842, indicating de-glucuronidation. The fragment at *m*/*z* 298.0678 (−1.7 ppm) was consistent with a homolytic cleavage of the bromine atom, as observed in the MS/MS spectrum of the parent. After 90 min hydrolysis with β-glucuronidase, the peak area of M1 remained basically unaltered. M1 is therefore likely a result of direct *N*-glucuronidation, probably at the pyrazole ring.

## 4. Discussion

### 4.1. In Silico Prediction

The metabolite forecast through in silico open-source tools may overestimate the number of biotransformational products. However, they can provide a holistic overview of potential biotransformation reactions of xenobiotics, which is of particular importance in the case of NPSs [34]. In this study, 23 metabolites were predicted through GLORYx with a threshold at 20% to avoid overpredicting flubrotizolam’s metabolites. However, only the two major metabolites detected in vitro were also predicted (M6 as pM1 and M5 as pM5), while the other 21 predicted metabolites were not detected in the incubates. This demonstrates that in silico predictions did not accurately anticipate flubrotizolam metabolism, highlighting the importance of experimental data, such as data on human hepatocyte incubation in metabolite identification studies. However, predictions can be used as a tool to optimize LC-HRMS/MS analyses and software-aided data mining, as illustrated in this study [20].

### 4.2. Incubation with Pooled Human Hepatocytes

The incubation of flubrotizolam with human hepatocytes and subsequent analysis using UHPLC-HRMS/MS and software-aided data mining resulted in the tentative identification and characterization of six metabolites. These metabolites were transformed mainly through phase I hydroxylation and further II glucuronidation reactions. The results obtained are consistent with flubrotizolam’s chloro-phenyl analog, brotizolam, and other similar thieno-triazolo benzodiazepines [29,32]. Based on our experimental study, identifying flubrotizolam and its hydroxylated metabolites, α-hydroxy- (M5) and 6-hydroxy-flubrotizolam (M6), in authentic biological samples should point to flubrotizolam intake, especially when this designer benzodiazepine is extensively metabolized in urine. This is juxtaposable to the proscribed markers, α- and 6-hydroxy-brotizolam, of its chloro-phenyl analog, which is principally metabolized by CYP3A4 isozymes [32,35].

In our study, flubrotizolam remained largely unmetabolized, as characterized by its high peak area after 3 h of incubation, and may indicate low clearance, which may prolong its pharmacological effects in vivo, thus affirming the QSAR results in Catalani et al.’s 2023 study [10]. The extrahepatic metabolism and high elimination of unmetabolized flubrotizolam cannot be excluded, although it would be inconsistent with brotizolam in vivo metabolism [32]. Furthermore, the overall activity of flubrotizolam could be enhanced if its main metabolites, α- and 6-hydroxyflubrotizolam, are active, which is not uncommon for benzodiazepines, although a scientific query may be necessary in order to assess their potential effects.

### 4.3. Limitations

This study compares LC-HRMS areas, which may vary with analytical conditions, such as the extraction method, mobile phase composition, and the ionization efficiency. Only a properly validated method using certified analytical standards can accurately inform on the relative abundance of flubrotizolam and its metabolites.

Incubation studies with hepatocytes containing necessary enzymes and cofactors have demonstrated comparable numbers and levels of metabolism products as in vivo studies, making the model a well-established first approach for NPS metabolites’ identification [16,17,18,36,37,38]. However, it will be important to analyze flubrotizolam-positive biospecimens to corroborate the current study. This will improve the interpretation and recommendation of consumption markers that may be useful in clinical and forensic cases. Notwithstanding, adapting the high-resolution fragmentation spectra of flubrotizolam and its metabolites in online databases will improve the identification of positive intoxication cases that can be used to further understand the drug’s pharmacokinetics.

## 5. Conclusions

Our approach enabled the identification and elucidation of the chemical structures of six flubrotizolam metabolites in this study. We thus propose flubrotizolam and its α- and 6-hydroxy metabolites as consumption markers. Adapting the HRMS spectra of these markers will be useful in improving authentic positive detections and help clinical and forensic toxicologists and legislators in curbing the abuse of this novel designer benzodiazepine.

## Figures and Tables

**Table 1:** Proposed elemental composition and biotransformation, retention time, accurate molecular mass, mass error (ppm), and the LC peak area of flubrotizolam and metabolites after 3 h of incubation in positive ionization mode (and negative ionization mode where detected).

ID	RT (min)	Biotransformation	Elemental Composition	[M + H]^+^ (*m*/*z*) [M − H]^−^ (*m*/*z*)	Mass Error, ∆ppm	Peak Area at T_3h_
M1	10.24	*N*-Glucuronidation (pyrazole)	C_21_H_19_BrFN_4_O_6_S	553.0195	0.42	8.5 × 10^5^
M2	11.21	Hydroxylation (pyrazole) + Reduction (pyrazole) + Glucuronidation	C_21_H_20_BrFN_4_O_7_S	571.0294 569.0165	0.20 3.10	4.1 × 10^6^ 2.4 × 10^6^
M3	12.15	Hydroxylation (diazepine) + *O*-Glucuronidation	C_21_H_18_BrFN_4_O_7_S	569.0138 567.0010	0.29 3.38	4.9 × 10^6^ 4.1 × 10^6^
M4	12.28	Hydroxylation (diazepine) + *O*-Glucuronidation	C_21_H_18_BrFN_4_O_7_S	569.0138 567.0010	0.29 3.38	1.9 × 10^6^ 1.8 × 10^6^
M5	13.43	Hydroxylation (pyrazole)	C_15_H_10_BrFN_4_OS	392.9816	0.13	2.8 × 10^7^
M6	13.70	Hydroxylation (diazepine)	C_15_H_10_BrFN_4_OS	392.9816	0.13	2.0 × 10^7^
Flubrotizolam	15.08	Parent	C_15_H_10_BrFN_4_S	376.9862	−1.15	1.3 × 10^9^

## Data Availability

The original contributions presented in the study are included in the article and Supplementary Materials, further inquiries can be directed to the corresponding author.

## Supplementary Materials

The following supporting information can be downloaded at: https://www.mdpi.com/article/10.3390/metabo14090506/s1. Figure S1: Flubrotizolam-detailed HRMS/MS fragmentation pathway; Figure S2: 6-Hydroxy-flubrotizolam (M6)-detailed HRMS/MS fragmentation pathway; Figure S3: α-Hydroxy-flubrotizolam (M5)-detailed HRMS/MS fragmentation pathway; Figure S4: α-Hydroxy-reduced flubrotizolam glucuronide (M2)-detailed HRMS/MS fragmentation pathway; Figure S5: Diazepine contraction of α-hydroxy metabolites in azolo-type benzodiazepines; Table S1: Inclusion lists for data-dependent MS/MS acquisition; Table S2: Potential transformations in Compound Discoverer workflow; Table S3: In silico-predicted metabolites, transformations, probability scores, and SMILES.
