# Supplementary material for: Exploring the Metabolism of Flubrotizolam, a Potent Thieno-Triazolo Diazepine, Using Human Hepatocytes and High-Resolution Mass Spectrometry
Source: Metabolites. 2024 Sep 19;14(9):506. doi: 10.3390/metabo14090506 (PMC11433749; doi:10.3390/metabo14090506)
Supplement: Supplementary file 1 [file metabolites-14-00506-s001.zip › metabolites-3175246-supplementary.pdf]

Table S1. ^{79}Br and ^{81}Br Inclusion List for Data Dependent MS/MS Acquisition

Transformation	Molecular Formula	$[M^{79}Br + H]^+$ m/z	$[M^{81}Br + H]^+$ m/z	$[M^{79}Br - H]^-$ m/z	$[M^{81}Br - H]^-$ m/z	Comments
Flubrotizolam	$C_{15}H_{10}BrFN_4S$	376.9866	378.9846	374.9721	376.9700	Parent
+O	$C_{15}H_{10}BrFN_4OS$	392.9815	394.9795	390.9670	392.9650	Oxidation [Hydroxylation] or Diazepine opening to aldehyde
-2H +2O	$C_{15}H_8BrFN_4O_2S$	406.9608	408.9588	404.9463	406.9442	Carboxylation
+2O	$C_{15}H_{10}BrFN_4O_2S$	408.9765	410.9744	406.9619	408.9599	Oxidation [Di-hydroxylation]
+6C +8H +7O	$C_{21}H_{18}BrFN_4O_7S$	569.0136	571.0116	566.9991	568.9970	Oxidation + Glucuronidation
+4O +S	$C_{15}H_{10}BrFN_4O_4S_2$	472.9384	474.9363	470.9238	472.9218	Oxidation + Sulfation
+H -Br	$C_{15}H_{11}FN_4S$	299.0761	-	297.0616	-	Debromination
+H -F	$C_{15}H_{11}BrN_4S$	358.9960	360.9940	356.9815	358.9795	Defluorination
+6C +9H +6O	$C_{21}H_{19}BrFN_4O_6S+$	553.0187	555.0167	-	-	*N*-Glucuronidation
+2H +O	$C_{15}H_{12}BrFN_4OS$	394.9972	396.9952	392.9826	394.9806	Diazepine opening to alcohol
+2H +2O	$C_{15}H_{12}BrFN_4O_2S$	410.9921	412.9901	408.9776	410.9755	Diazepine opening 2 or Dihydrodiol formation
-2H +3O	$C_{15}H_8BrFN_4O_3S$	422.9557	424.9537	420.9412	422.9391	Oxidation + Carboxylation
-C -2H	$C_{14}H_8BrFN_4S$	362.9710	364.9689	360.9564	362.9544	Demethylation
+2C +3H +O	$C_{17}H_{13}BrFN_4OS+$	418.9972	420.9952	-	-	*N*-Acetylation
+6C +8H +8O	$C_{21}H_{18}BrFN_4O_8S$	585.0085	587.0065	582.9940	584.9920	Oxidation [Di-hydroxylation] + Glucuronidation
+H -Br +O	$C_{15}H_{11}FN_4OS$	315.0710	-	313.0565	-	Debromination + Oxidation
+H -F +O	$C_{15}H_{11}BrN_4OS$	374.9910	376.9889	372.9764	374.9744	Defluorination + Oxidation
+6C +9H -Br +7O	$C_{21}H_{19}FN_4O_7S$	491.1031	-	489.0886	-	Debromination + Oxidation + Glucuronidation
+6C +9H -F +7O	$C_{21}H_{19}BrN_4O_7S$	551.0231	553.0210	549.0085	551.0065	Defluorination + Oxidation + Glucuronidation
+2H -F -Br	$C_{15}H_{12}N_4S$	281.0855	-	279.0710	-	Defluorination + Debromination
+3H -Br +2O	$C_{15}H_{13}FN_4O_2S$	333.0816	-	331.0670	-	Debromination + Dihydrodiol
+3H -F +2O	$C_{15}H_{13}BrN_4O_2S$	393.0015	394.9995	390.9870	392.9849	Defluorination + Dihydrodiol
+H -Br +2O	$C_{15}H_{11}FN_4O_2S$	331.0660	-	329.0514	-	Debromination + Oxidation [Di-hydroxylation]
+H -F +2O	$C_{15}H_{11}BrN_4O_2S$	390.9859	392.9838	388.9713	390.9693	Defluorination + Oxidation [Di-hydroxylation]
+2H -Br -F +O	$C_{15}H_{12}N_4OS$	297.0805	-	295.0659	-	Defluorination + Debromination + Oxidation
+2H +3O	$C_{15}H_{12}BrFN_4O_3S$	426.9870	428.9850	424.9725	426.9704	Diazepine opening 2 + Oxidation
+2C +4H +3O	$C_{17}H_{14}BrFN_4O_3S$	453.0027	455.0006	450.9881	452.9861	Diazepine opening 2 + *N*-Acetylation
+10C +16H -Br +3N +6O +S	$C_{25}H_{26}FN_7O_6S_2$	604.1443	-	602.1297	-	Glutathione conjugation [Bromine]

+10C +16H -F +3N +6O +S	$C_{25}H_{26}BrN_7O_6S_2$	664.0642	666.0622	662.0497	664.0476	Glutathione conjugation [Fluorine]
+C +2H +O	$C_{16}H_{12}BrFN_4OS$	406.9972	408.9952	404.9826	406.9806	Oxidation + Methylation
+10C +16H -Br +3N +7O +S	$C_{25}H_{26}FN_7O_7S_2$	620.1392	-	618.1246	-	Oxidation + Glutathione conjugation [Bromine]
+10C +16H -F +3N +7O +S	$C_{25}H_{26}BrN_7O_7S_2$	680.0591	682.0571	678.0446	680.0425	Oxidation + Glutathione conjugation [Fluorine]
-C -2H +O	$C_{14}H_8BrFN_4OS$	378.9659	380.9639	376.9513	378.9493	Oxidation + Demethylation
-C -2H +2O	$C_{14}H_8BrFN_4O_2S$	394.9608	396.9588	392.9463	394.9442	Oxidation [Di-hydroxylation] + Demethylation

Table S2. Transformations for generating potential phase I and phase II metabolites of flubrotizolam in Compound Discoverer data mining software

Phase I	Desaturation (H_2 →)<br>Dihydrodiol formation (→ H_2 O_2)<br>Ketone formation (O → H_2)<br>Oxidation (→ O)<br>Oxidative Deamination to Alcohol (H_2N → HO)<br>Oxidative Debromination (Br → HO)<br>Oxidative Defluorination (F → HO)<br>Reduction (→ H_2)<br>Reduction Debromination (Br → H)<br>Reduction Defluorination (F → H)
Phase II	Acetylation (H → C_2 H_3 O)<br>Cysteine conjugation on Br (Br → C_3 H_6 N O_2 S)<br>Cysteine conjugation on F(F → C_3 H_6 N O_2 S)<br>Cysteine-Glycine Conjugation on Br (Br → C_5 H_9 N_2 O_3 S)<br>Cysteine-Glycine Conjugation on F (F → C_5 H_9 N_2 O_3 S)<br>Glucuronide Conjugation (H → C_6 H_9 O_6)<br>GSH Conjugation on Br (Br → C_{10} H_{16} N_3 O_6 S)<br>GSH Conjugation on F (F → C_{10} H_{16} N_3 O_6 S)<br>Sulfation (H → H O_3 S)
Max # Dealkylation	2
Max # Phase II	2
Max # of Reactions	5

Table S3. Biotransformation, elemental structure, probability score and Simplified Molecular-Input Line Entry Specification (SMILES) of flubrotizolam metabolites predicted using GLORYx free-base webtool.

ID	Transformation	Structure	Score (%)	SMILES
Parent	Flubrotizolam	$C_{15}H_{10}BrFN_4S$	-	Fc1ccccc1C1=NCc2nnc(C)n2c2sc(Br)cc21
pM1	Hydroxylation (Diazepine ring)	$C_{15}H_{10}BrFN_4OS$	40	OC1N=C(c2cc(Br)sc2n2c(C)nnc21)c1ccccc1F
pM1-1	+ *O*-Sulfation	$C_{15}H_{10}BrFN_4O_4S$	30	O=S(O)(=O)OC1N=C(c2cc(Br)sc2n2c(C)nnc21)c1ccccc1F
pM2	*N*-Dealkylation	$C_{15}H_{10}BrFN_4OS$	40	O=Cc1nnc(C)n1c1sc(Br)cc1C(=N)c1ccccc1F
pM3	*N*-Oxidation	$C_{15}H_{11}BrFN_4OS^+$	40	O[N+]=1Cc2nnc(C)n2c2sc(Br)cc2C=1c1ccccc1F
pM4	Carboxylation	$C_{15}H_8BrFN_4O_2S$	39	OC(=O)c1nnc2CN=C(c3cc(Br)sc3n12)c1ccccc1F
pM4-1	+ *O*-Glucuronidation	$C_{21}H_{16}BrFN_4O_8S$	26	O=C(OC1OC(O)C(O)C(C(=O)O)C1O)c1nnc2CN=C(c3cc(Br)sc 3n12)c1ccccc1F
pM5	Hydroxylation (Pyrazole)	$C_{15}H_{10}BrFN_4OS$	39	OCc1nnc2CN=C(c3cc(Br)sc3n12)c1ccccc1F
pM5-1	+ *O*-Sulfation	$C_{15}H_{10}BrFN_4O_4S_2$	34	O=S(O)(=O)OCc1nnc2CN=C(c3cc(Br)sc3n12)c1ccccc1F
pM5-2	+ *O*-Glucuronidation	$C_{21}H_{18}BrFN_4O_7S$	26	OC1OC(OCc2nnc3CN=C(c4cc(Br)sc4n23)c2ccccc2F)C(O)C(C(O)=O)C1O
pM6	Oxidation (Sulfide)	$C_{15}H_{10}BrN_4OS$	32	O=S1C=2n3c(C)nnc3CN=C(c3ccccc3F)C=2C=C1Br
pM7	Hydroxylation (Aromatic)	$C_{15}H_{10}BrFN_4OS$	28	Oc1cc(C2=NCc3nnc(C)n3c3sc(Br)cc32)c(F)cc1
pM7-1	+ *O*-Glucuronidation	$C_{21}H_{18}BrFN_4O_7S$	26	OC1OC(Oc2cc(C3=NCc4nnc(C)n4c4sc(Br)cc43)c(F)cc2)C(O)C (C(=O)O)C1O
pM7-2	+ *O*-Sulfation	$C_{15}H_{10}BrFN_4O_4S_2$	25	O=S(O)(=O)Oc1cc(C2=NCc3nnc(C)n3c3sc(Br)cc32)c(F)cc1
pM7-3	+ *O*-Methylation	$C_{16}H_{12}BrFN_4OS$	24	COc1cc(C2=NCc3nnc(C)n3c3sc(Br)cc32)c(F)cc1
pM8	Hydroxylation (Aromatic)	$C_{15}H_{10}BrFN_4OS$	28	Oc1cc(F)c(cc1)C1=NCc2nnc(C)n2c2sc(Br)cc21
pM8-1	+ *O*-Glucuronidation	$C_{21}H_{18}BrFN_4O_7S$	26	OC1OC(C(O)=O)C(O)C(Oc2cc(F)c(cc2)C2=NCc3nnc(C)n3c3sc (Br)cc32)C1O
pM8-2	+ *O*-Methylation	$C_{16}H_{12}BrFN_4OS$	26	COc1cc(F)c(cc1)C1=NCc2nnc(C)n2c2sc(Br)cc21
pM8-3	+ *O*-Sulfation	$C_{15}H_{10}BrFN_4O_4S_2$	26	O=S(O)(=O)Oc1cc(F)c(cc1)C1=NCc2nnc(C)n2c2sc(Br)cc21
pM9	Hydroxylation (Aromatic)	$C_{15}H_{10}BrFN_4OS$	28	Oc1cccc(C2=NCc3nnc(C)n3c3sc(Br)cc32)c1F
pM9-1	+ *O*-Glucuronidation	$C_{21}H_{18}BrFN_4O_7S$	26	OC1OC(C(=O)O)C(O)C(Oc2cccc(C3=NCc4nnc(C)n4c4sc(Br)cc 43)c2F)C1O
pM9-2	+ *O*-Sulfation	$C_{15}H_{10}BrFN_4O_4S_2$	25	O=S(O)(=O)Oc1cccc(C2=NCc3nnc(C)n3c3sc(Br)cc32)c1F
pM9-3	+ *O*-Methylation	$C_{16}H_{12}BrFN_4OS$	24	COc1cccc(C2=NCc3nnc(C)n3c3sc(Br)cc32)c1F
pM10	Glutathione Conjugation	$C_{25}H_{28}BrFN_7O_6S_2$	20	O=C(NCC(O)=O)C(NCC(N)CCC(O)=O)CSc1cc2c(s1)n1c(CN= C2c2ccc(O)cc2F)nnc1C

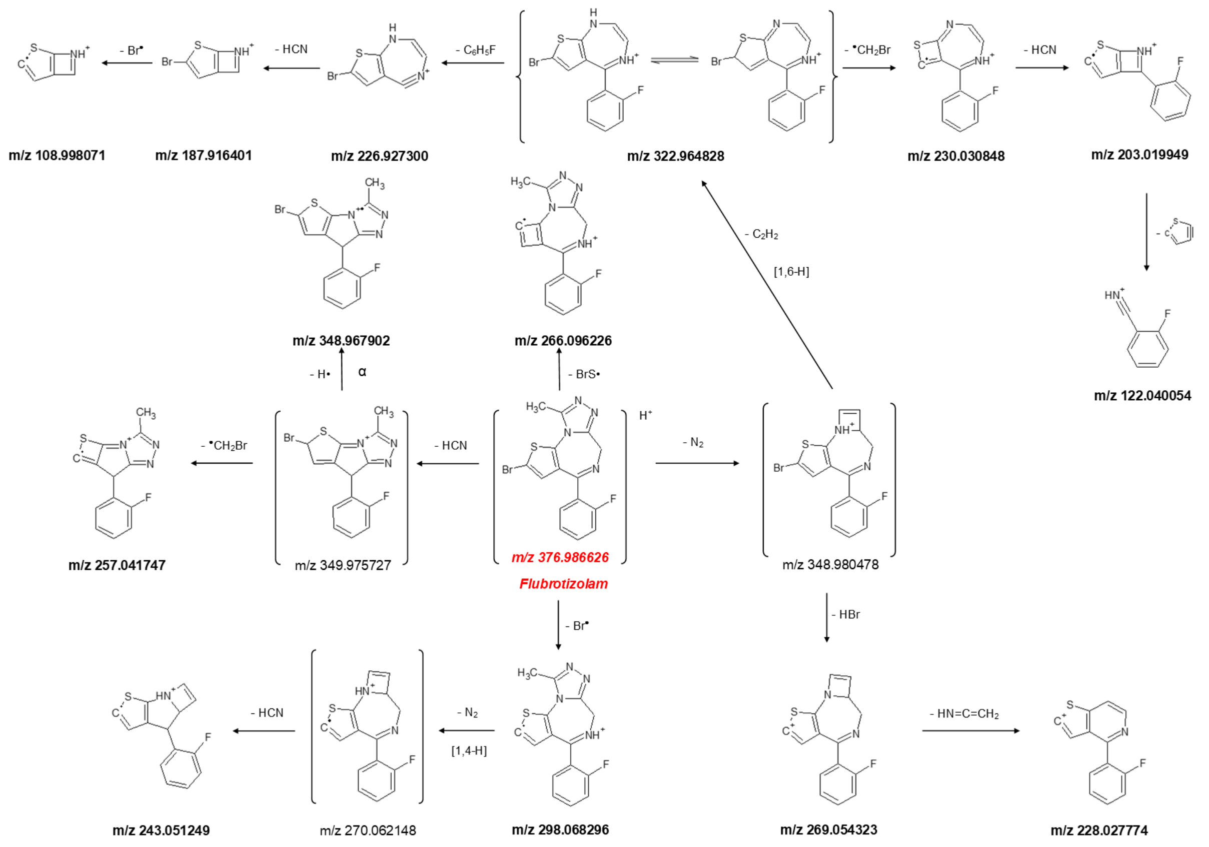

Supplementary Figure S1. Proposed HRMS/MS fragmentation pathway for flubrotizolam. *m/z* in **BOLD** appear in the MS/MS spectrum of flubrotizolam

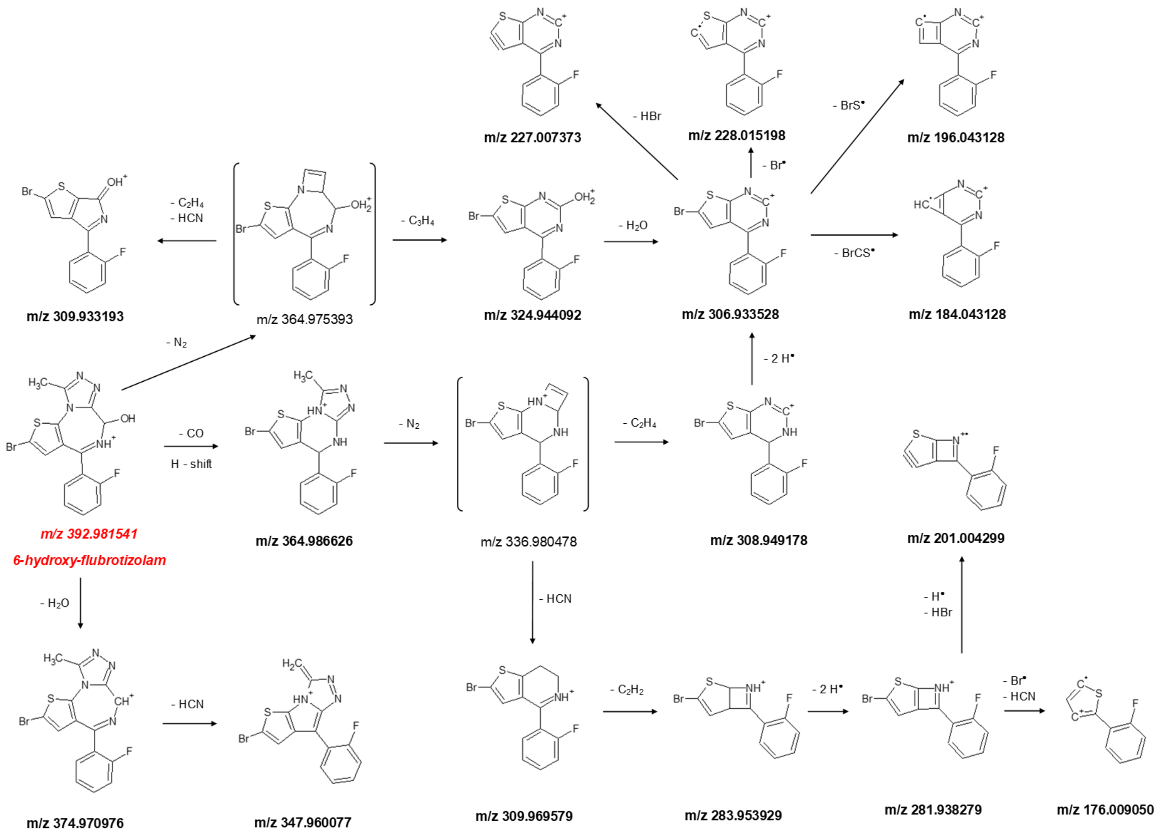

Supplementary Figure S2. Proposed HRMS/MS fragmentation pathway for 6-hydroxy-flubrotizolam (M6).

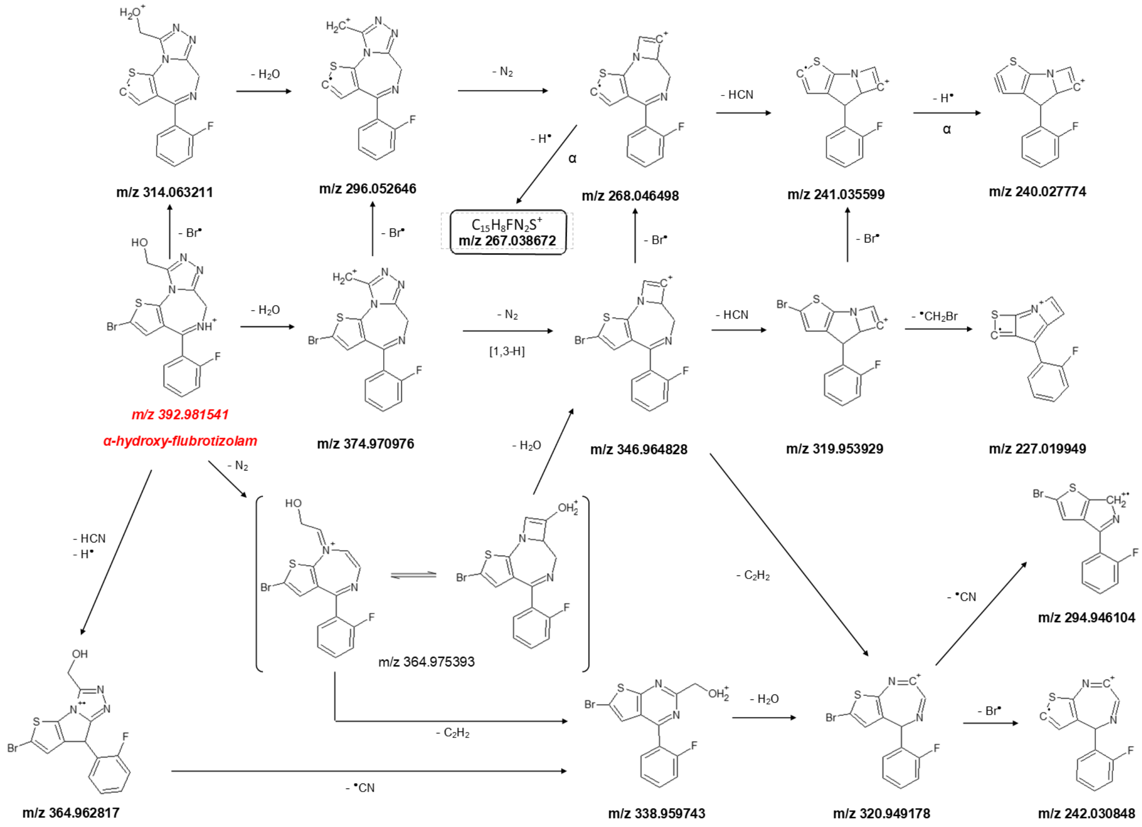

Supplementary Figure S3. Proposed HRMS/MS fragmentation pathway for α-hydroxy flubrotizolam (M5).

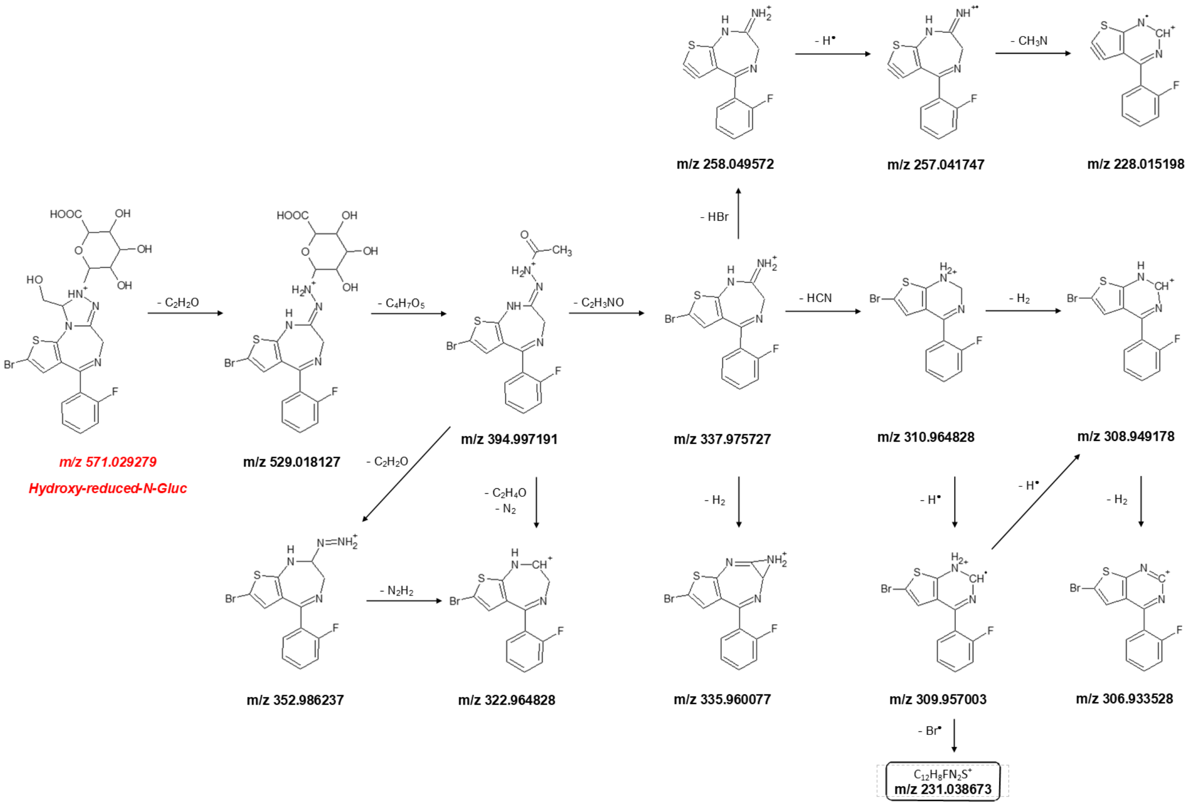

Supplementary Figure S4. Proposed HRMS/MS fragmentation pathway for f α-hydroxy-reduced flubrotizolam glucuronide (M2).

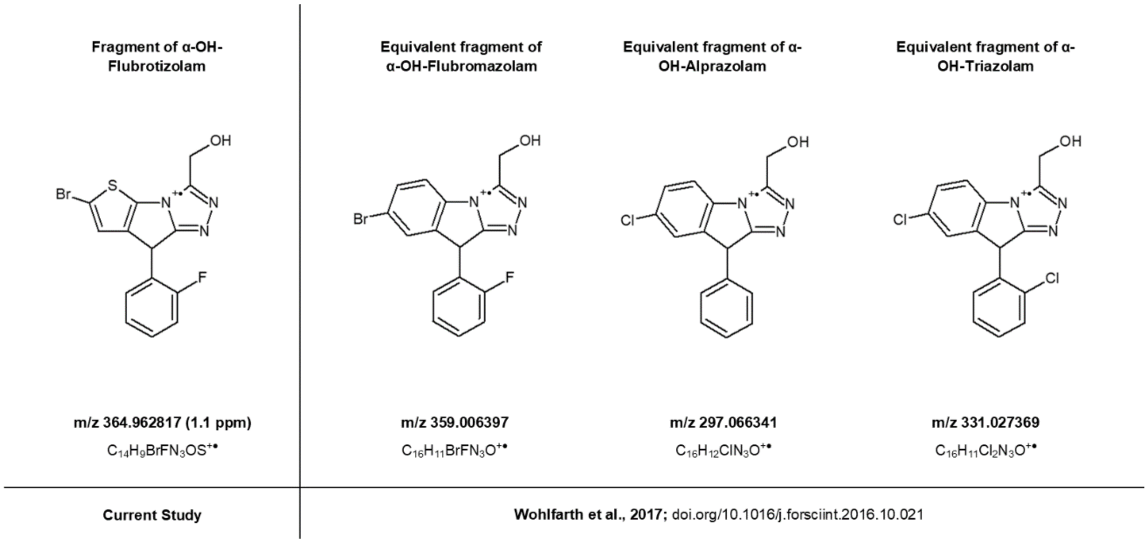

Supplementary Figure S5. Diazepine ring contraction of α-hydroxy metabolites in azolo-type benzodiazepines.
